# Physical correlation between stochasticity and process-induced damage in ferroelectric memory devices

**DOI:** 10.1186/s40580-025-00505-1

**Published:** 2025-08-29

**Authors:** Ryun-Han Koo, Seungwhan Kim, Jiseong Im, Sangwoo Ryu, Kangwook Choi, Sung-Ho Park, Jonghyun Ko, Jongho Ji, Mingyun Oh, Jangsaeng Kim, Gyuweon Jung, Sung-Tae Lee, Daewoong Kwon, Wonjun Shin, Jong-Ho Lee

**Affiliations:** 1 Department of Electrical and Computer Engineering and Inter- University Semiconductor Research Center (ISRC), Seoul National University, Seoul, 08826 Korea; 2 Department of Electronic Engineering, Sogang University, Seoul, 04107 Republic of Korea; 3 School of Electronic and Electrical Engineering, Hongik University, Seoul, 04066 Republic of Korea; 4 Department of Electrical Engineering, Hanyang University, Seoul, 04763 Korea; 5 Department of Semiconductor Convergence Engineering, Sungkyunkwan University, Suwon, 16419 Republic of Korea

**Keywords:** Stochasticity, Ferroelectric tunnel junction, Low-frequency noise (LFN), Trap formation, Plasma-induced damage

## Abstract

This study investigates the influence of sputtering plasma-induced damage on stochastic characteristics in HfZrO₂ (HZO)-based ferroelectric tunnel junctions (FTJs), with an emphasis on memory and neuromorphic device optimization. Variation of the sputtering plasma power during top electrode deposition introduces distinct levels of trap within the HZO layer. Low-frequency noise (LFN) spectroscopy and temperature-dependent electrical measurements confirm that higher plasma power generates additional shallow-level traps, thereby promoting Poole-Frenkel conduction while simultaneously increasing current noise magnitude. Although the resulting enhancements in on-current density and ferroelectric tunnel electroresistance (TER) ratio are beneficial for high-density memory integration, these conditions also elevate stochastic fluctuations, potentially degrading read margins and long-term endurance. Furthermore, the observed increase in stochasticity negatively affects neuromorphic inference accuracy, particularly after endurance cycling stress. These results demonstrate the critical interplay among plasma process conditions, trap density, and LFN in FTJs. By systematically engineering sputtering process parameters, we optimize the electrical performance with minimized stochastic noise. This approach provides guidelines for the development of next-generation ferroelectric-based memories and neuromorphic systems with consideration of stochasticity, where robust performance and reliability are imperative for large-scale integration.

**Graphical abstract:**

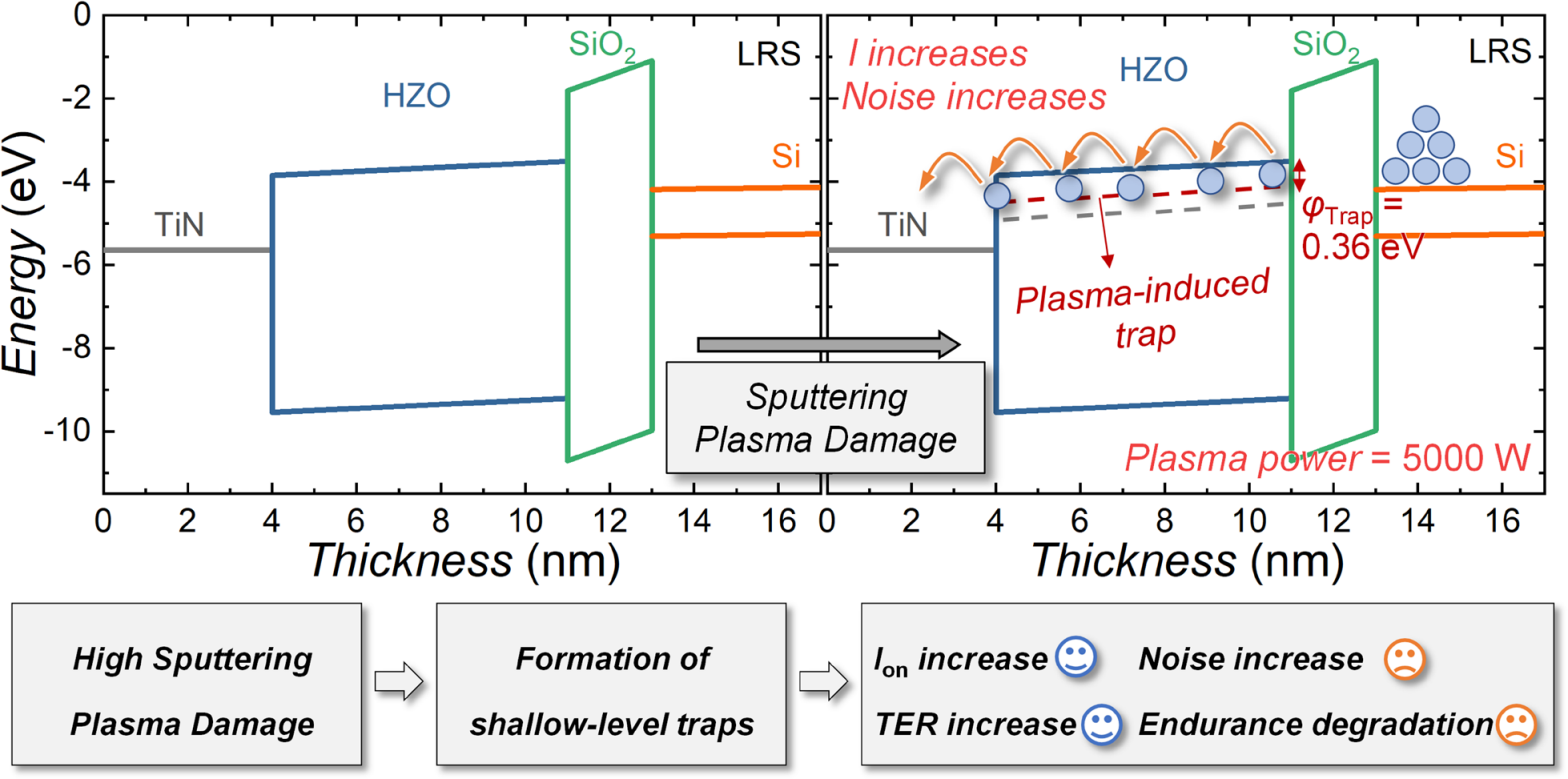

## Introduction

As the semiconductor industry pushes device miniaturization and performance to unprecedented levels, extreme manufacturing environments—characterized by high temperatures, large mechanical stresses, and aggressive plasma-based deposition processes—have become integral to the fabrication of next-generation electronics [1–3]. Under these extreme conditions, advanced memory architectures must not only achieve high density and fast access but also maintain robust endurance against repeated switching and stress cycles, all while withstanding extreme fabrication and operating conditions [4–7]. This convergence of device scaling and harsh processing conditions underscores the need for manufacturing-optimized strategies that ensure both reliability and functionality at the nanoscale [8–12].

Among various emerging memories, ferroelectric-based devices, particularly HfO_2_-ZrO_2_ (HZO)-based ferroelectrics, are considered prime candidates for next-generation memory and computing architectures due to their compatibility with standard complementary metal-oxide-semiconductor (CMOS), low-voltage operation, and robust retention [13–15]. However, fabricating these devices often involves a complex series of processes—such as sputtering plasma deposition at high power levels that can introduce physical damage and tensile strain into the ultrathin ferroelectric layers during the annealing process [16]. During high-temperature deposition or annealing, energetic ions and neutrals from plasma can lead to the creation of defects, material phase changes, and deterioration of interfaces. In subsequent device operation, especially in high-endurance cycling scenarios (e.g., more than 104 times of program/erase pulses), these process-induced defects can accumulate, undermining the reliability and scaling potential of ferroelectric memory and neuromorphic devices alike [17–19].

To date, several studies have reported on ferroelectric switching mechanisms and the broad advantages of HZO systems, including their inherent endurance, nondestructive readout, and high-speed polarization switching [20–25]. Nevertheless, many of these investigations focused on device physics aspects—such as ferroelectric domain dynamics or conduction mechanisms—largely neglecting the extreme process conditions and the resultant impact on stochastic reliability [26–28]. In real-world manufacturing lines, sputtering damage is a critical concern: increased plasma power can expedite deposition throughput but also intensify bombardment of the ferroelectric thin film, creating additional oxygen vacancies (Vₒ) or shallow traps that degrade long-term performance and induce read noise [29–32]. Such process-induced damage becomes especially pivotal for neuromorphic applications, where repeated synaptic weight updates and massive parallel operations necessitate robust and predictable electrical characteristics [33–35].

Neuromorphic accelerators using ferroelectric tunnel junctions (FTJs) rely on precise conductance tuning for vector-matrix multiplications (VMM) [36–38]; however, random telegraph noise or 1/*f* noise can significantly distort output accuracy, particularly in downscaled geometries [39–41]. Understanding how the manufacturing parameters, e.g., plasma power, drive trap formation and degradation is thus crucial for optimizing both memory endurance and neuromorphic inference accuracy. Among various electrical characterization techniques, low-frequency noise (LFN) spectroscopy offers unique advantages due to its high sensitivity to microscopic defects and trapping phenomena within electronic devices. Specifically, LFN provides a powerful, non-destructive method for quantitatively evaluating defect distributions, energy levels, and trap densities, which significantly affect device reliability and performance. Due to these advantages, LFN analysis has been extensively applied across diverse electronic platforms, ranging from logic devices such as conventional Si-based semiconductors, organic semiconductors, and two-dimensional (2D) materials [42–47], to memory devices including resistive random-access memories (RRAM) and ferroelectric-based memories [48–60]. By accurately probing defect-related fluctuations, LFN spectroscopy enables direct insights into defect states and conduction mechanisms, establishing itself as an essential tool for reliability studies in various semiconductor and memory device architectures.

In this work, we explore the interplay between sputtering plasma-induced damage and the stochastic behavior in HZO-based FTJs. Specifically, we systematically vary the plasma power during top electrode deposition and examine its effect on trap densities, endurance fatigue, and noise spectra under high-temperature and high-cycling conditions. Low-frequency noise (LFN) spectroscopy and temperature-dependent measurements reveal how process-driven defects alter the carrier transport mechanism via shallow-level trap formation, resulting in amplified read noise despite potentially increasing the on-current and tunneling electroresistance (TER). We further demonstrate that such plasma-induced damage can have dual implications in neuromorphic systems: while higher plasma power can yield larger on/off ratios in the pristine state—beneficial for conductance tuning—it also exacerbates noise and reliability decay under extended endurance cycling, ultimately degrading inference accuracy. By correlating manufacturing conditions with both device-level physics and system-level performance, this study provides practical guidelines for extreme manufacturing of ferroelectric memories. Our results underscore that careful optimization of sputtering parameters is essential to balance throughput, device performance, and reliability. Ultimately, these findings contribute to the design of resilient, high-endurance ferroelectric memory and neuromorphic hardware capable of thriving in the extreme manufacturing regimes of future semiconductor technologies.

## Results and discussion

### Fabrication process and electrical characteristics

**Fig. 1:**
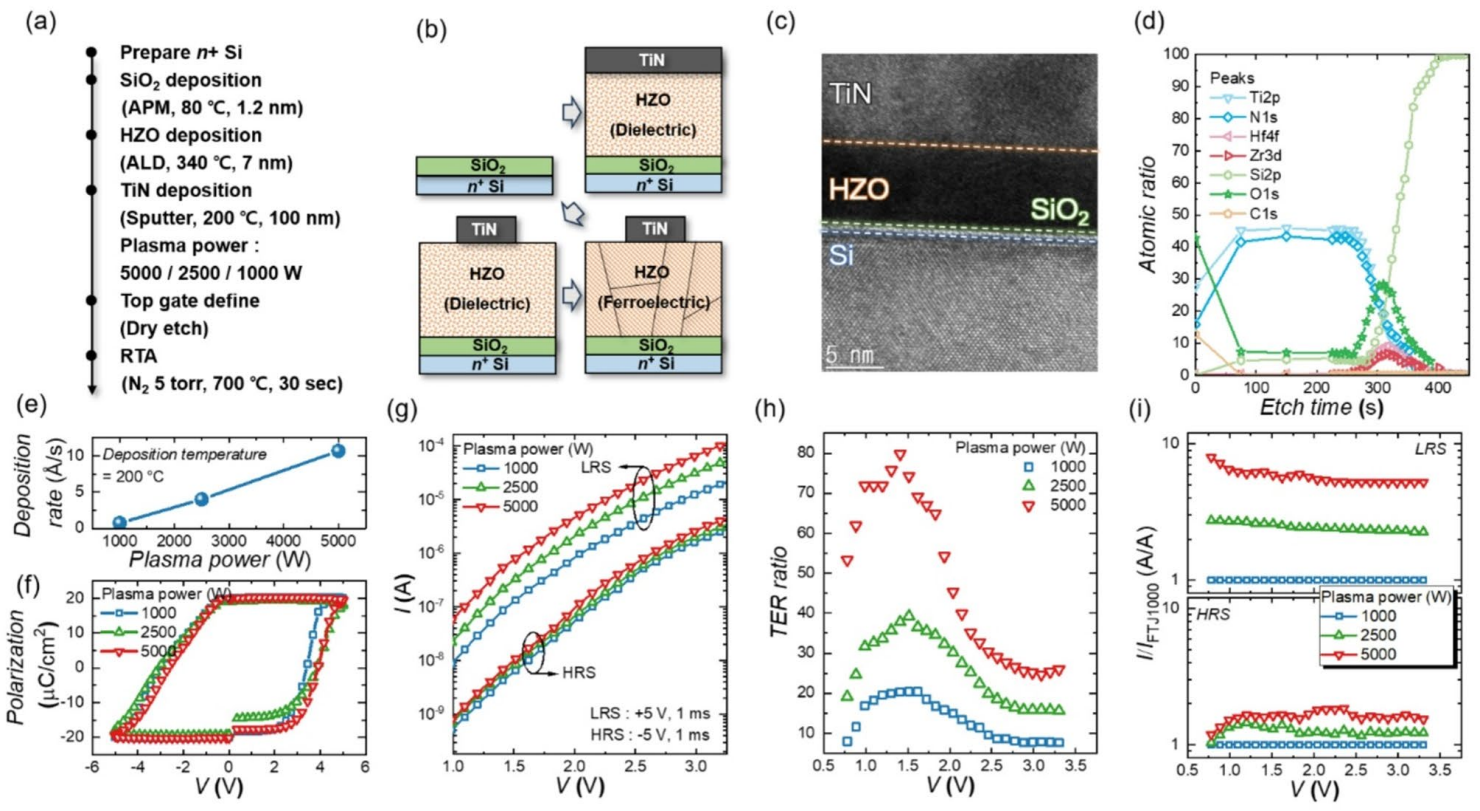
(**a**) Fabrication process of the FTJ. (**b**) Schematic diagram of the fabrication process. (**c**) Cross-sectional TEM image of the fabricated FTJ. (**d**) Atomic ratio detected during depth XPS analysis versus etch times. (**e**) Deposition rate of TiN under different plasma power levels (1000 W, 2500 W and 5000 W). (**f**) *P*-*V* curves of FTJs with varying plasma power levels. (**g**) *I*-*V* curves in the LRS and HRS of FTJs with different plasma damage. (**h**) TER ratio versus V for FTJs under different plasma power levels. (**i**) Normalized current in the LRS (upper) and HRS (lower) of FTJs with varying plasma power

Figure 1(a) shows the fabrication process of the FTJ used in this study. The bottom electrode is single-crystalline silicon, highly doped with *n*^+^ (resistivity < 0.005 Ω·cm). A 1.2 nm SiO₂ insulator layer is grown using a chemical oxidation process at 80 °C in an APM solution (NH₃OH: 1 L, H₂O₂: 1 L, H₂O: 5 L) for 10 min. Subsequently, a 7 nm HZO ferroelectric layer is deposited via thermal atomic layer deposition (ALD) at 340 °C. The supercycle consists of one cycle of Hf and one cycle of Zr, repeated 50 times (growth per cycle: 1.4 nm). The top electrode is a 100 nm TiN layer deposited using a sputtering process at 200 °C [61]. To investigate the effects of plasma power, sputtering plasma power is split into 1000, 2500, and 5000 W.

Note that the deposition time is adjusted to achieve a uniform 100 nm thickness of the metal despite the varying deposition rates at different plasma power levels (1000 W: 720 s, 2500 W: 360 s, 5000 W: 180 s). To clearly isolate and assess the impact of RF sputtering power alone, all other deposition parameters were strictly maintained constant across all conditions. Specifically, the substrate temperature was consistently held at 200 °C, and the Ar/N_2_ gas flow rates were maintained at 15/85 sccm for every deposition run. Top gate patterning is performed through a photolithography process, followed by dry etching of the TiN layer to define the top gate. The rapid thermal annealing process (N₂ ambient, 5 Torr, 30 s, 700 °C) induces ferroelectricity in the HZO layer by transitioning its stable phase from m-phase (dielectric) to o-phase (ferroelectric), caused by the difference in the thermal expansion coefficients between TiN and the underlying HZO layer [39]. A schematic diagram of the fabrication process is shown in Fig. 1(b).

Figure 1(c) shows the cross-sectional transmission electron microscope (TEM) image of the fabricated FTJ. The thicknesses of the HZO and SiO₂ layers (7 nm and 1.2 nm, respectively) are confirmed via TEM images. Figure 1(d) shows the depth X-ray photoelectron spectroscopy (XPS) analysis of the fabricated FTJ. Atomic peaks are analyzed as a function of etch time, showing that Ti2p and N1s ratios increase during the initial etch stage (0–100 s). Subsequently, Hf4f, Zr3d, O1s, and Si2p peaks, corresponding to the HZO and SiO₂ layers, emerge between 270–320 s. At etch times exceeding 400 s, the Si2p signal from the bottom electrode becomes dominant. These results confirm that the fabricated FTJ successfully forms the intended TiN-HZO-SiO₂-Si structure characteristic of a metal-ferroelectric-insulator-semiconductor (MFIS) structure. Figure 1(e) shows the deposition rate of TiN as a function of plasma sputtering power. The deposition rate increases with higher plasma power due to an elevated ion density and ion collision energy, which enhance the etching rate of the metal target and increase the deposition flux onto the substrate. However, higher plasma power also increases the energy of ions striking the substrate, potentially inducing physical damage to the device. This damage can affect the electrical characteristics of the device, such as read noise, on-current density, and tunnel electroresistance (TER) ratio.

**Fig. 2:**
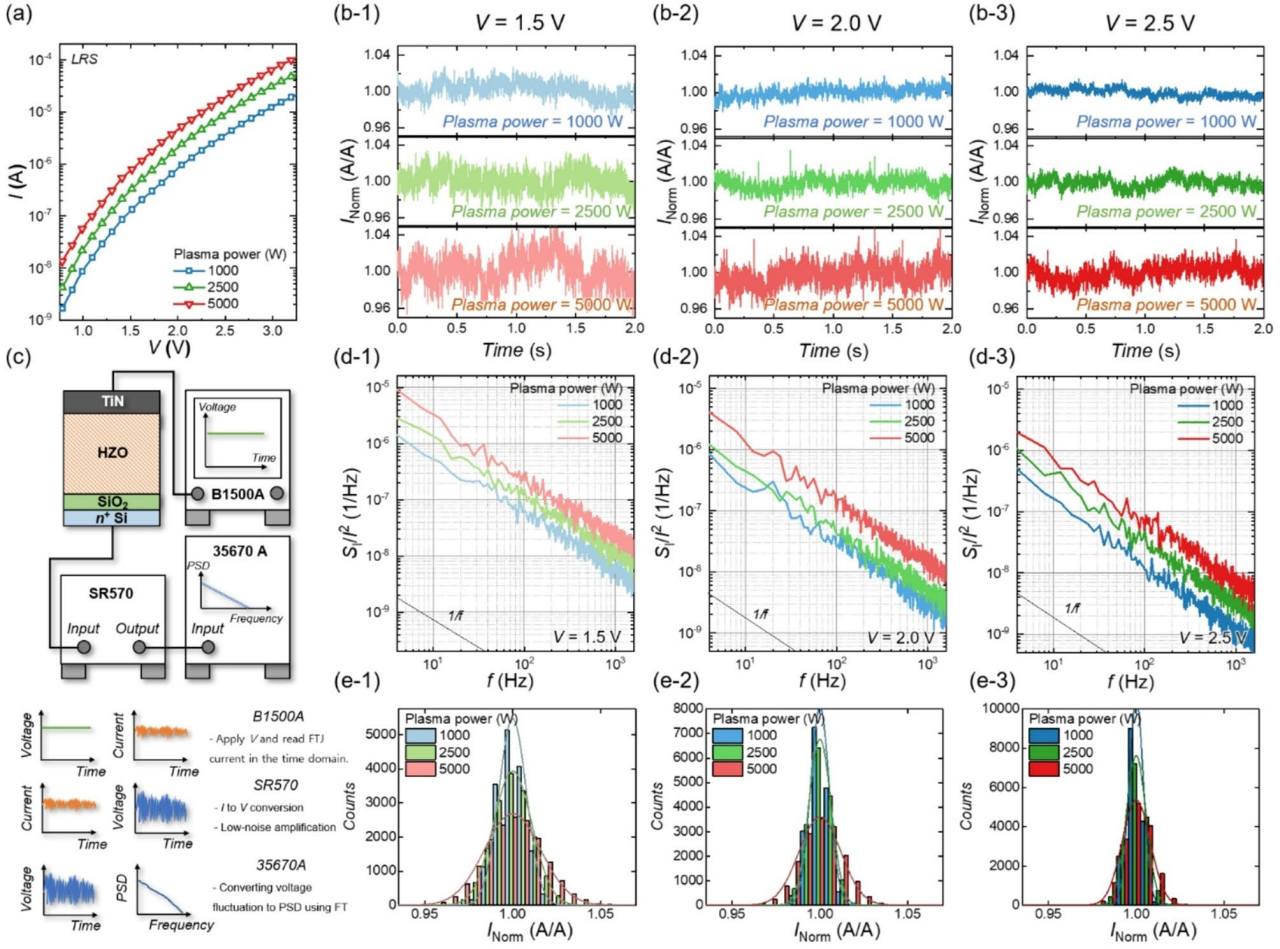
(**a**) *I*-*V* curves of FTJs in the LRS under different plasma power levels. (**b**) *I*_norm_ sampled at various *V*s: (b-1) *V* = 1.5 V, (b-2) *V* = 2.0 V, and (b-3) *V* = 2.5 V, for FTJs with different plasma power levels. (**c**) Experimental setup for LFN spectroscopy. (**d**) *S*_I_/*I*^2^ of FTJs measured at various *V*s: (d-1) *V* = 1.5 V, (d-2) *V* = 2.0 V, and (d-3) *V* = 2.5 V under different plasma power levels. (**e**) Frequency distribution histogram of *I*_norm_

Figure 1(f) shows the polarization-voltage (*P*-*V*) curves for FTJs fabricated under different plasma power conditions. The remnant polarization (*P*_r_) and coercive field (*E*_c_) exhibit negligible differences across the plasma power levels, indicating that ferroelectricity is largely unaffected by plasma power. Figure 1(g) shows the current-voltage (*I-V*) characteristics of FTJs fabricated under varying plasma power levels. A 5 V, 1 ms pulse is applied to the top electrode to induce the low resistance state (LRS), while a -5 V, 1 ms pulse induces the high resistance state (HRS), with the bottom electrode (*n*^+^-Si) grounded. The FTJs demonstrate distinct memory states—HRS and LRS—determined by the switching direction of ferroelectric dipoles, leading to different TER values. Interestingly, despite minimal differences in ferroelectric characteristics (Fig. 1(f)), the *I*-*V* characteristics exhibit significant differences. Higher plasma power leads to increased current levels in both LRS and HRS, with the increase being more pronounced in the LRS.

Figure 1(h) shows the TER ratio as a function of *V* for FTJs fabricated under different plasma power conditions. The TER ratio, defined as the resistance ratio between the HRS and the LRS, is a key performance metric for FTJs. A higher TER ratio improves the distinction between the two states, enhancing read margin and enabling better area scalability. As shown in Fig. 1(g), while both LRS and HRS currents increase with higher plasma power, the LRS current increase is more pronounced, resulting in an improved TER ratio. Figure 1(h) further confirms that FTJs fabricated with higher plasma power consistently exhibit larger TER ratios across the entire voltage range (0.8 to 3.2 V).

Figure 1(i) shows the normalized current values of FTJs in the low resistance state (LRS, upper panel) and high resistance state (HRS, lower panel), normalized to the current of the FTJ fabricated at 1000 W (FTJ1000). While the HRS shows minimal current variation across different plasma power levels, the LRS exhibits significant changes. These results indicate that plasma power-induced damage primarily affects the current conduction mechanism in the LRS. Note that the observed differences in current behavior with plasma power clearly indicate spatially confined plasma-induced damage. Specifically, because the top TiN electrode is deposited after the 7 nm-thick HZO film has been fully crystallized, the sputtering plasma primarily interacts with the upper surface of the capacitor stack. Consequently, the plasma-induced damage is naturally localized near the TiN/HZO interface.

### Impact of plasma power on stochasticity

To analyze the underlying physical mechanisms by which plasma power affects the current conduction mechanism of FTJs in the LRS, the stochasticity of FTJs is examined across different plasma power levels.

Figure 2(a) shows the *I*-*V* curves of FTJs in the LRS under varying plasma power conditions. Figure 2(b) presents transient current sampling results for FTJs in the LRS at different read *V*: (b-1) *V* = 1.5 V, (b-2) *V* = 2.0 V, and (b-3) *V* = 2.5 V, sampled at a frequency of 3200 Hz for 2 s. To compare the magnitude of read noise, the *y*-axis is normalized by the mean current value (*I*_norm_​), with all *y*-axis ranges unified to 0.95–1.05.

Across all plasma power levels, the read noise decreases as the read *V* increases, consistent with the typical LFN behavior in semiconductor devices: higher voltages generate larger currents, increasing the number of charge carriers involved and reducing the normalized current fluctuation. However, an abnormal trend is also observed: as the plasma power used during top gate sputtering increases, the read noise stochasticity also increases, despite the current level rising (Fig. 2(a)). This deviation from the typical LFN behavior in the FTJs suggests that plasma power-induced modifications change key noise-generation sources, such as trap density, trap energy levels, and trapping-detrapping time constants. This finding highlights the need for further investigation into how these parameters influence the observed stochastic behavior.

**Fig. 3:**
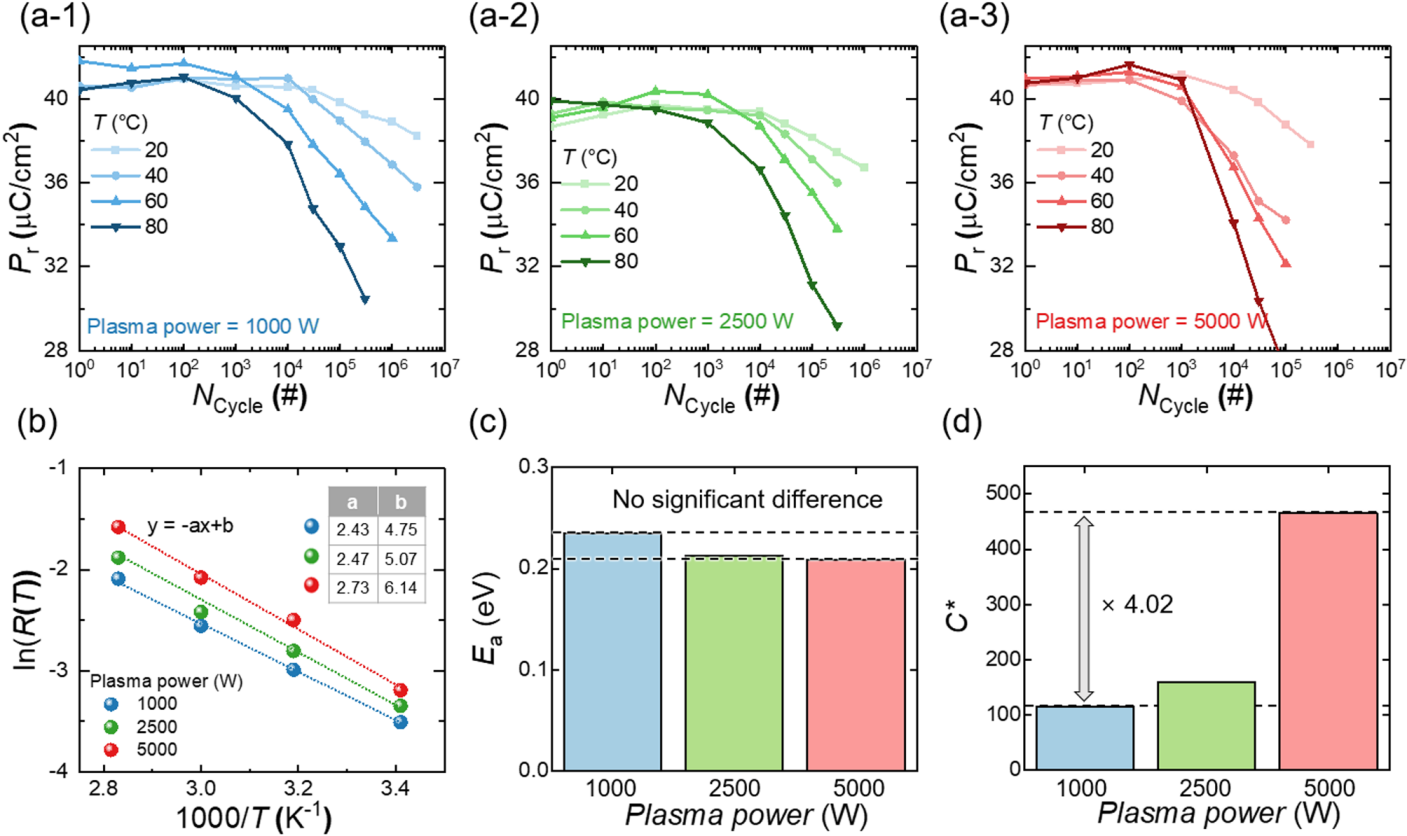
(**a**) Cycling endurance characteristics of FTJs fabricated under plasma powers of (a-1) 1000 W, (a-2) 2500 W, and (a-3) 5000 W at different temperatures. (**b**) ln*R*(*T*) versus 1000/*T* curves with linear fitting results (dotted lines) for FTJs fabricated with different plasma power levels. (**c**) *E*_a_ of FTJs as a function of plasma power. (d) *C*^∗^ for FTJs as a function of plasma power

LFN spectroscopy is employed to provide an in-depth analysis of stochasticity in FTJs. This technique is a non-destructive and reliable method for probing microscopic processes in physical devices. In electronic devices, LFN typically originates from mechanisms such as charge carrier trapping and detrapping at traps, generation-recombination processes, and mobility fluctuations. The spectral characteristics of LFN, often expressed as 1/*f*^α^ (where *f* is the frequency), are highly sensitive to underlying physical phenomena and material properties, making LFN spectroscopy an effective tool for evaluating device reliability and performance.

Figure 2(c) shows the detailed experimental setup used for LFN spectroscopy. The drain current was routed through shielded triaxial cabling into a Stanford SR570 low-noise current pre-amplifier, biased using a Keysight B1500A semiconductor parameter analyzer. The resulting amplified voltage signal was then analyzed by an Agilent 35,670 A FFT analyzer to obtain the power spectral density (PSD). Noise measurements were performed at bias conditions ranging from 1.5 to 2.5 V for the LRS and the HRS. Each PSD spectrum reported herein was derived by averaging 20 sequential PSD segments, each measured over a 0.25 s interval at a sampling rate of 3.2 kHz, yielding a total acquisition time of 5 s per spectrum. The system noise floor was verified using a dummy resistor before each measurement run.

The current fluctuation $\:\varDelta\:I\left(t\right)$ is defined as [62]:

1 $$\:\varDelta\:I\left(t\right)=I\left(t\right)\:-\:\stackrel{-}{I}\:\:\:\:\:\:\:\:\:\:\:$$

where $\:I\left(t\right)$ is the instantaneous current at time *t* and $\:\stackrel{-}{I}\:$ is the average current over time. The PSD quantifies the power of current fluctuations as a function of frequency and is calculated as [43]:

2 $$\:{S}_{I}\left(f\right)=\:\underset{T\to\:\infty\:}{\text{lim}}{\frac{1}{T}\left|{\int\:}_{-T/2}^{T/2}\varDelta\:I\left(t\right){e}^{-j2\pi\:ft}\:dt\right|}^{2}$$

where $\:{S}_{I}\left(f\right)$ is the PSD of current fluctuations at *f*, *T* is the total observation time, and *j* is the imaginary unit. The $\:{S}_{I}\left(f\right)$ provides the distribution of fluctuation power across frequencies, enabling analysis of noise characteristics. To account for the influence of the absolute current magnitude, the PSD is normalized by the square of the average current ($\:{\stackrel{-}{I}}^{2}$), represented as *S*_I_/*I*^2^.

**Fig. 4:**
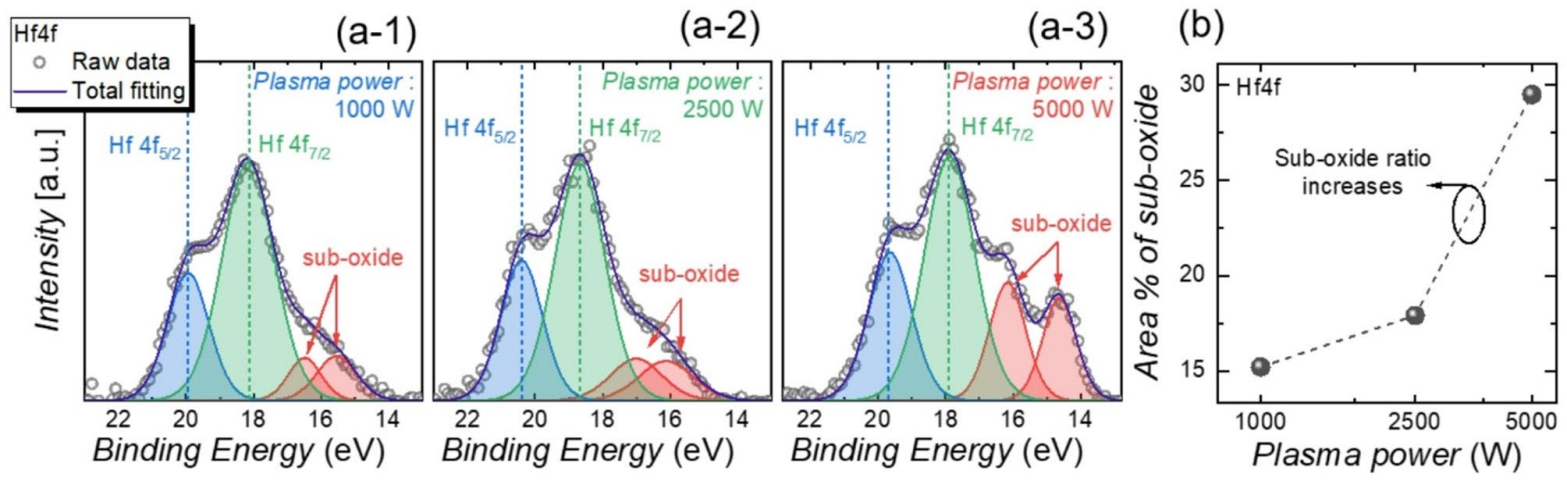
(**a**) XPS spectra of Hf 4f core-level scans for FTJs fabricated with plasma powers of (a-1) 1000 W, (a-2) 2500 W, and (a-3) 5000 W, showing the relative intensities of the main peaks and sub-oxide components. (**b**) Area percentage of sub-oxide binding extracted from the Hf 4f spectra as a function of plasma power

Figure 2(d) shows the *S*_I_/*I*^2^ as a function of *f* for FTJs fabricated with different plasma powers and measured under various voltage biases: (d-1) *V* = 1.5 V, (d-2) *V* = 2.0 V, and (d-3) *V* = 2.5 V. The normalized PSD decreases with increasing V for a given plasma power but increases with higher plasma power at a fixed V. These trends align with the time-domain results in Fig. 2(b), confirming that the observed current noise originates from fluctuations of intrinsic physical quantities in the device rather than from external noise sources. Under all measured voltage and plasma power conditions, the *S*_I_/*I*^2^ follows a 1/*f* noise trend. This behavior typically results from the superposition of multiple 1/*f*^2^ Lorentzian noise sources with varying corner frequencies, where each source contributes to the overall spectrum at different frequency ranges. The consistent appearance of 1/*f* noise across all tested conditions suggests the presence of a common noise generation mechanism related to the physical properties of the device. However, the magnitude of the observed 1/*f* noise varies with plasma power, indicating that plasma-induced damage affects parameters such as trap density, trap energy levels, and trapping-detrapping dynamics [63, 64]. A detailed analysis later clarifies how plasma power modulates these noise-related parameters.

Figure 2(e) shows the *I*_norm_ for FTJs under different voltage biases (sampling rate = 3200 Hz, sampling time = 5 s). The measured currents are fitted to a Gaussian normal distribution (represented as a line), further validating the 1/*f* noise behavior [65]. Similar to Figs. 2(b) and 2(d), the sigma of the normalized transient current increases with plasma power, despite the increase in current level. Note that this increase in read noise adversely affects both memory and neuromorphic system performance, emphasizing the need for its thorough analysis and control.

The consistent results from time-domain measurements (Figs. 2(b) and 2(e)) and frequency-domain analysis (Fig. 2(d)) demonstrate that plasma power significantly increases current stochasticity, suggesting that plasma-induced damage alters key physical parameters affecting the carrier transport mechanism. Further analysis, including the extraction of degradation *E*_a_ and trap energy levels, is conducted to elucidate the relationship between plasma power and stochasticity.

### Impact of plasma power on degradation activation energy

Analyzing the degradation mechanism provides critical insights into the defect characteristics of devices. To examine the impact of plasma power on the degradation mechanism of FTJs, endurance characteristics are evaluated over a wide temperature range (*T* = 20, 40, 60, and 80 °C). Figures 3(a) shows the cycling endurance test results of FTJs fabricated with varying plasma powers: (a-1) 1000 W, (a-2) 2500 W, and (a-3) 5000 W. Across all plasma power levels, degradation accelerates with increasing *T*. Additionally, at the same *T*, higher plasma power results in faster degradation. Note that given the short pulse duration and thin ferroelectric barrier (7 nm), Joule heating generated during cycling is efficiently dissipated between pulses [48]. Additionally, the stable chuck temperature throughout testing ensures minimal temperature drift.

Based on the temperature-dependent degradation trends, the activation energy for degradation (*E*_a_) of each FTJ is extracted using the following equations [66, 67]:

3 $$ P\left( N \right) = {P_0}\left[ {1 - R\left( T \right)logN} \right] $$

where ***N*** is the number of applied cycling damage pulses, *P*(*N*) is the *P*_r_ after the *N*-th cycle, *P*₀ is the initial *P*_r_, and *R*(*T*) is the fatigue rate dependent on temperature. The fatigue rate *R*(*T*) is further defined as [66, 67]:

4 $$ R\left( T \right) = C*\,exp\left[ { - {E_a}/{k_B}T} \right] $$

where C* is the degradation coefficient, *E*ₐ is the activation energy for degradation, and *k*_B_ is the Boltzmann constant (8.617 × 10⁻⁵ eV/K).

**Fig. 5:**
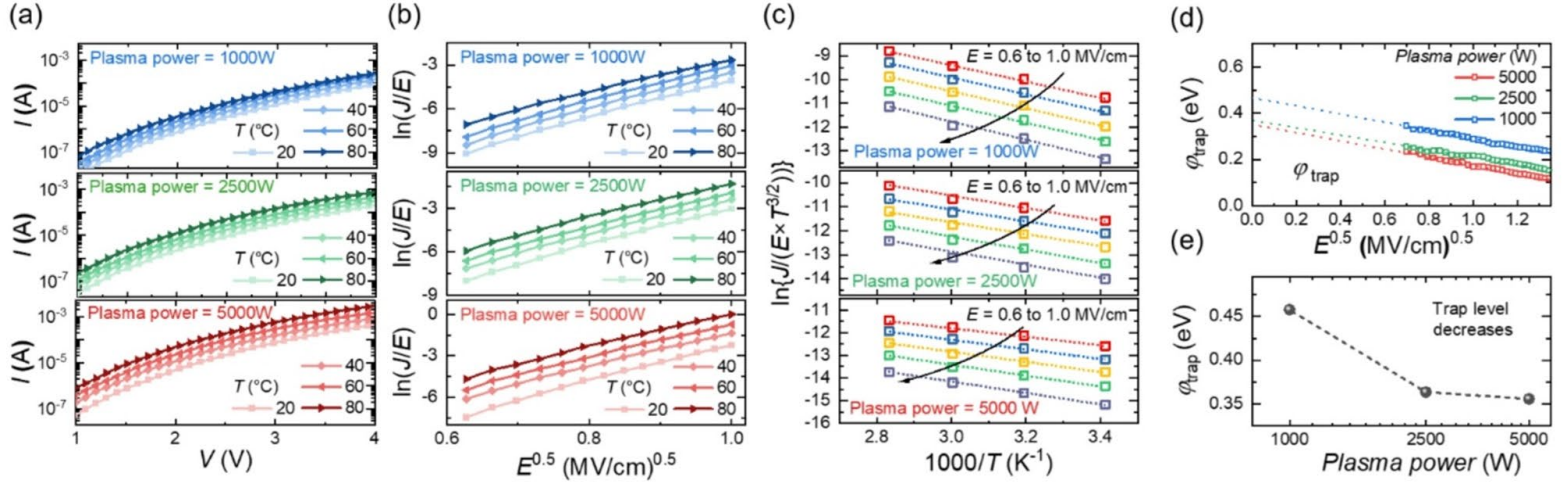
(**a**) *I*-*V* curves of FTJs measured at different temperatures. (**b**) ln(*J*/*E*) versus *E*^0.5^ curves of FTJs at different temperatures. (**c**) ln(*J*/(*E*⋅*T*^3/2^)) versus 1000/*T* curves with linear fitting results (dotted lines). (**d**) ϕ_trap_ versus *E*^0.5^ for FTJs fabricated under different plasma power levels. (**e**) Extracted intrinsic ϕ_trap_s for FTJs with varying plasma power levels

Figure 3(b) shows the ln[*R*(*T*)] versus 1000/*T* curves and their linear fitting results (dotted lines) for FTJs fabricated under different plasma power levels. The inset table in Fig. 3(b) provides the slope (*a*) and *y*-intercept (*b*) values of the linear fitting lines. According to Eq. (4), the slope of the ln[*R*(*T*)] versus 1000/*T* curve (*a*) is proportional to *E*ₐ, while the *y*-intercept (*b*) is proportional to C*. The slope shows negligible variation across plasma power levels, while the *y*-intercept changes significantly with plasma power. Figures 3(c) and 3(d) show the extracted *E*ₐ and C* values, respectively, for FTJs fabricated at different plasma powers. As shown in Fig. 3(c), *E*ₐ remains nearly constant regardless of plasma power, indicating that the degradation mechanism represented by *E*ₐ is unaffected by plasma power. However, as shown in Fig. 3(d), C*, which reflects the initial state vulnerability to degradation, increases significantly with plasma power. Notably, the FTJ fabricated at 5000 W exhibits a C* value approximately four times higher than that of the FTJ fabricated at 1000 W. This finding indicates that while plasma power does not affect the fundamental degradation mechanism represented by *E*ₐ, it leads to an initial state more susceptible to degradation, as indicated by the higher C*. Thus, the occurrence of process-induced damage due to plasma exposure during top electrode deposition is clearly demonstrated.

### Impact of plasma power on carrier transport mechanism and noise

To further investigate the nature of process-induced damage due to plasma exposure, depth XPS analysis is performed on FTJs fabricated with top electrodes sputtered at different plasma power levels. After sputtering the top electrode (TiN) with varying plasma damage levels, the TiN layer is removed via wet etching (H₂O₂: 2 L, H₂O: 8 L, 80 °C). The exposed HZO layer is then subjected to depth XPS analysis using an ion gun energy of 4000 eV. For Hf 4f core level analysis, the binding energy is calibrated using the hydrocarbon (C-H) signal in the C1s peak at 284.8 eV. A Shirley background correction is applied within a range from 23.0 eV to 13.0 eV. The intensity is normalized to the peak maximum of the Hf 4f_7/2_ curve, and Gaussian functions are used for two doublets to analyze the *V*ₒ concentration [68].

The Hf 4f peaks for the HZO layer are observed at binding energies of 20.3 − 19.7 eV and 18.7 − 17.9 eV, corresponding to the Hf 4f_5/2_ and Hf 4f_7/2_ spectra, respectively. Sub-oxide peaks attributed to the HfO₂ phase, resulting from defects such as *V*ₒ and interstitial compounds, are identified in the ranges 17.0 eV- 16.1 eV and 15.8 eV- 14.7 eV. The relative intensities of each peak are considered during the fitting process [68]. Figures 4(a) show the XPS spectra of the Hf 4f scan for FTJs with top electrodes sputtered at plasma powers of 1000 W (a-1), 2500 W (a-2), and 5000 W (a-3). Compared to FTJs fabricated at 1000 W and 2500 W, those fabricated at 5000 W exhibit reduced intensities for the Hf 4f_5/2_ and Hf 4f_7/2_ peaks, while the sub-oxide peak intensities are notably higher. Figure 4(b) plots the area percentage of sub-oxide binding as a function of plasma power. The sub-oxide intensity increases with plasma power, indicating a higher concentration of *V*_o_. This result suggests that the increased initial fatigue rate is linked to elevated oxygen vacancy concentrations induced by higher plasma power during the fabrication process.

The *V*_o_ generated by plasma damage significantly affects the carrier transport mechanism, contributing to read noise and explaining the increased stochasticity in FTJs. Figure 5(a) shows the *I*-*V* curves at different *T*s. As *T* increases, the *I* also increases at the same *V*. In FTJs with a thick insulator stack (7 nm HZO), the current conduction mechanism commonly follows Poole-Frenkel (P-F) emission, a bulk-limited process. When the conduction mechanism follows P-F emission, the relationship between current density (*J*) and electric field (*E*) is expressed as [69]:

**Fig. 6:**
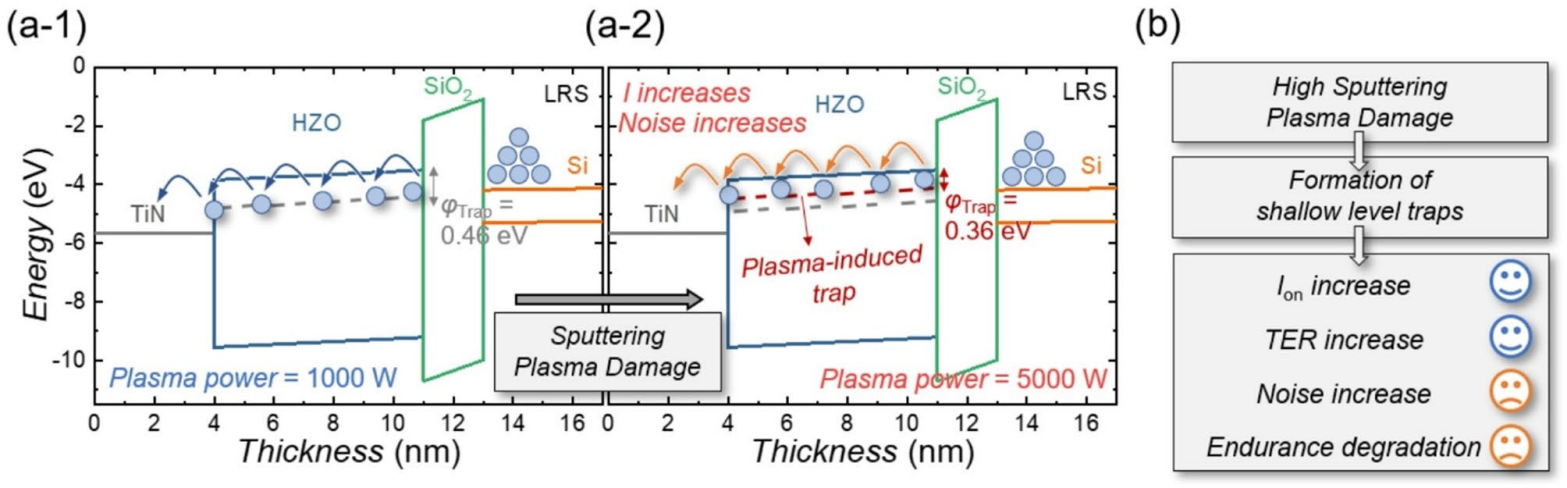
(**a**) Schematic diagrams illustrating the carrier transport mechanism and noise generation process in FTJs fabricated under different plasma power levels: (a-1) 1000 W and (a-2) 5000 W. (**b**) Summary of key performance metrics for FTJs with high plasma power-induced damage

5 $$\:J/E=q\mu\:{N}_{c}\text{exp}[-\frac{q\left({\phi\:}_{trap}-\sqrt{\frac{qE}{\pi\:{\epsilon\:}_{i}{\epsilon\:}_{0}}}\right)}{kT}]$$

where *q* is the elementary charge (1.6 × 10⁻¹⁹ C), *µ* is the electron mobility, *N*_c_ is the effective density of states in the conduction band, and *E* is the electric field of HZO. *φ*_trap_ is the trap energy level, which is the energy required for an electron to escape from a trap state, ϵ_i_ is the relative permittivity of insulator, ϵ_0_ is the permittivity of free space (8.854 × 10^− 12^ F/m). From Eq. (5), when carrier conduction follows P-F emission, ln(*J*/*E*) is proportional to *E*^0.5^. Figure 5(b) shows the ln(*J*/*E*) versus *E*^0.5^ curves for FTJs fabricated at different plasma powers and *T*s. The linear relationship across all temperatures confirms that the LRS current conduction mechanism of the fabricated FTJs follows P-F emission, independent of plasma power. This result aligns with findings from previous studies. By plotting ln(*J*/(*E*⋅*T*^3/2^)) versus 1000/*T*, the slope of the resulting curve can be used to extract the *φ*_trap_. Figure 5(c) shows the ln(*J*/(*E*⋅T^3/2^)) versus 1000/*T* plot, demonstrating a clear linear fit. Figure 5(d) plots the *φ*_trap_ as a function of *E*^0.5^ based on the slope obtained from Fig. 5(c). The effective *φ*_trap_ varies with the external electric field, and the *y*-intercept at *E*^0.5^ = 0 represents the bulk *φ*_trap_ of the HZO layer in the absence of an external electric field.

Figure 5(e) shows the intrinsic trap energy levels extracted for each plasma power based on the *y*-intercept from Fig. 5(d). The results indicate that higher plasma power introduces additional traps at shallower energy levels within the HZO layer, causing carrier conduction to occur predominantly through these shallow traps.

When electron transport occurs via P-F emission, the current noise due to trapping-detrapping at P-F source traps follows the relationship [70]:

6 $$\:\frac{{{S_{IT}}}}{{{I_T}^2}} = \frac{{k{N_{T,HZO}}}}{{E{\epsilon ^2}A}}\frac{1}{f}$$

where *k* is the fitting parameter, *N*_T_​,_HZO_ is the trap density in HZO layer, *E* is the applied electric field, and ***A*** is the tunneling area. According to Eq. (6), noise from P-F emission follows a 1/*f* trend and inversely depends on the applied *E*. This behavior explains why, as shown in Fig. 2(d), *S*_I_/*I* ² consistently decreases with increasing *V* for a given plasma power. Additionally, conduction mechanisms such as direct tunneling (flat spectrum) and Fowler–Nordheim tunneling (field-insensitive 1/*f* noise) were systematically excluded based on their fundamental mismatch with our experimentally observed frequency- and field-dependent trends. Instead, the measured temperature-dependent and electric-field-dependent 1/*f* noise characteristics aligned precisely with Poole–Frenkel emission, clearly identifying plasma-induced shallow traps as the dominant intrinsic noise source. Furthermore, P-F emission explains the simulaneous increase in both time-domain and frequency-domain noise with higher plasma power, despite the concurrent rise in current levels. While *S*_I_/*I*² decreases with increasing *E* due to stronger electric fields reducing the relative impact of trapping-detrapping fluctuations, it increases with HZO trap density (*N*_T, HZO_), as a higher density of traps causes more frequent trapping-detrapping events, leading to higher noise. The degradation coefficients and depth-XPS analysis presented in Figs. 3 and 4 consistently suggest the occurrence of process-induced *V*_o_ due to increased plasma damage. Thus, higher plasma power during top electrode deposition generates additional shallow-level traps in the HZO layer. As sputtering power increases, a greater number of shallow traps form, leading to increased current due to enhanced Poole-Frenkel emission while also causing more frequent trapping-detrapping events, thereby increasing stochasticity. This correlation elucidates the relationship between plasma power, FTJ current conduction mechanisms, and the resulting read current stochasticity.

**Fig. 7:**
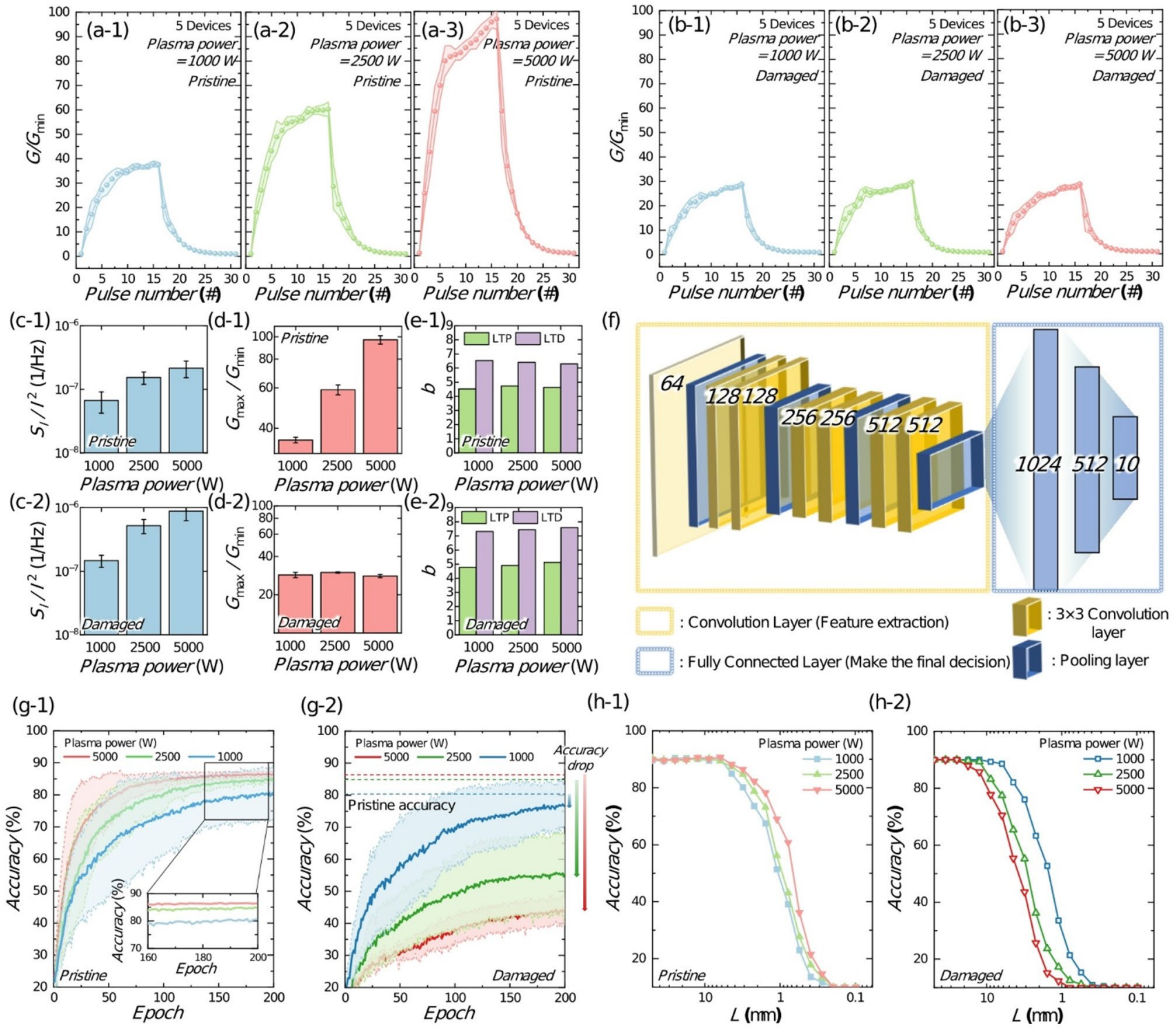
LTP–LTD characteristics measured from five FTJ devices under (**a**) pristine and (**b**) damaged (cycled to 10^5^ pulses) conditions. Data are presented as averaged curves (solid lines) with shaded areas indicating min–max variations across devices. (**c**) Averaged S_I_/I^2^ versus frequency obtained from five FTJ devices under pristine (c-1) and damaged (c-2) conditions, respectively, including min–max error bars. (**d**) Average values of Gmax/Gmin measured from five FTJ devices under pristine (d-1) and damaged (d-2) conditions, respectively, with min–max error bars indicating sample-to-sample variation. (**e**) β of FTJs measured in (e-1) pristine and (e-2) damaged states under different plasma power levels. (**f**) Schematic diagram of VGG11. (**g**) Accuracy as a function of epoch for (g-1) pristine and (g-2) damaged FTJs. (**h**) Accuracy versus FTJ area in the (h-1) pristine and (h-2) damaged FTJs

Figures 6(a) show a band diagram schematically illustrating the carrier transport mechanism in FTJs under different plasma power conditions. At lower plasma power (1000 W), traps responsible for P-F emission are located approximately 0.46 eV below the HZO conduction energy level (*E*_c, HZO_). However, at higher plasma power (5000 W), significant plasma damage induces the formation of additional *V*_o_, increasing the number of shallow traps. Consequently, the trap responsible for P-F emission moves closer to the conduction band, positioned 0.36 eV below *E*_c, HZO_, resulting in increased current magnitude. This occurs because carriers can more easily overcome the reduced energy barrier presented by the shallower traps, facilitating carrier transport through the HZO layer. Furthermore, the higher trap density resulting from increased plasma power causes more frequent trapping-detrapping events, raising the overall noise level. This interplay between current increase and noise generation explains the unique phenomenon where both current magnitude and noise rise simultaneously.

Figure 6(b) summarizes the experimental findings. High sputtering plasma power leads to the formation of a greater number of shallower traps within the HZO layer. As a result, LRS current and the TER ratio improve due to enhanced carrier conduction through these shallow traps. However, the increased trap density contributes to higher noise levels and degraded endurance characteristics, highlighting the critical trade-off between improved memory performance and increased stochasticity caused by plasma-induced damage.

### Effect of sputtering damage on neural network performance

High on-current and TER ratios are desirable characteristics for enhancing read margin and array scalability in FTJs. However, high noise levels and degradation caused by initial damage are undesirable properties. To evaluate the trade-offs resulting from sputtering-induced damage and its implications for neuromorphic systems, Python-based simulations are performed using the experimentally measured characteristics of the fabricated FTJs.

For FTJs to operate effectively as synapses in hardware-based neuromorphic systems, they need to exhibit long-term potentiation (LTP) and long-term depression (LTD) behavior in response to continuous pulse inputs, mimicking synaptic plasticity observed in biological neural networks [71, 72]. Figure 7(a) shows the LTP-LTD curves measured in the pristine state of FTJs sputtered with plasma powers of 1000 W (a-1), 2500 W (a-2), and 5000 W (a-3), where *G* denotes conductance, *G*_min_ represents minimum conductance, and *G*_max_ represents maximum conductance. Note that LTP is induced by applying a + 5 V, 100 µs pulse, while LTD is induced by applying a − 5 V, 100 µs pulse sequentially.

Figure 7(a) shows that as the sputtering power increases, the *G*_max_/*G*_min_ in the LTP-LTD characteristics becomes larger, expanding the representable conductance ratio. This occurs because the TER ratio increases with higher sputtering power, as shown in Fig. 1(h). Figure 7(b) shows the LTP-LTD curves of FTJs after cycling degradation for plasma powers of 1000 W (b-1), 2500 W (b-2), and 5000 W (b-3). Degradation is applied by repeatedly applying + 5 V, 1 ms and − 5 V, 1 ms pulses 10⁵ times. After degradation, the TER ratio decreases across all plasma power levels, reducing the *G*_max_/*G*_min_ range in the LTP-LTD process. The reduction is more pronounced for FTJs fabricated at higher plasma power levels, as they degrade more rapidly due to increased initial damage, as shown in Fig. 3(a). We note that the measured LTP–LTD characteristics shown in Fig. 7(a) and (b) exhibit monotonic conductance updates for at least the first 16 pulses, indicating a resolution of at least 16 effective conductance levels suitable for synaptic applications. Although adaptive pulse programming schemes such as incremental step pulse programming (ISPP) could potentially increase resolution further, we intentionally chose the simpler constant-pulse approach for practical applicability and alignment with our study’s focus on sputtering-induced damage effects [71].

In addition to LTP-LTD characteristics, read noise-induced stochasticity also critically affects the performance of neuromorphic systems. Since VMM operations in neuromorphic systems rely on Kirchhoff’s law to represent output values as current, read noise can distort computation results, degrading overall system performance.

To incorporate these factors into the simulation, Figs. 7(c–e) present the measured *S*_*I*_/*I*² (c), *G*_max_/*G*_min_ (d), and the nonlinearity factor (*β)* (e) for pristine and cycling-damaged FTJs. *β* is obtained by the following equations [71]:

7 $$\:{G}_{\text{L}\text{T}\text{P}}=B(1-\text{e}\text{x}\text{p}(-\frac{P}{{\beta\:}_{\text{L}\text{T}\text{P}}}\left)\right)\:+\:{G}_{min}\:\:$$

8 $$\:{G}_{\text{L}\text{T}\text{D}}=-B(1-\text{e}\text{x}\text{p}(\frac{P-{P}_{max}}{{\beta\:}_{\text{L}\text{T}\text{D}}}\left)\right)\:+\:{G}_{max}$$

9 $$\:B=({G}_{max}-{G}_{min})/(1-\text{e}\text{x}\text{p}\left(\frac{-{P}_{max}}{{A}_{\text{L}\text{T}\text{P},\text{L}\text{T}\text{D}}}\right))$$

where $\:{G}_{\text{L}\text{T}\text{P}}$ and $\:{G}_{\text{L}\text{T}\text{D}}$ are the conductance values during LTP and LTD, respectively. *P* and *P*_max_ denote the number of applied pulses and the maximum number of pulses, respectively. *β*_LTP_ and *β*_LTD_ are non-linearity factors associated with conductance updates in potentiation and depression. The constant *B* normalizes the conductance range based on *G*_min_, *G*_max,_ and *P*_max_. Figures 7(c–e) show the *S*_I_/*I*² increases after cycling damage for all plasma power levels due to the increased trap density. *G*_max_/*G*_min_ increases with plasma power in the pristine state but decreases after damage, converging to similar values across all plasma power levels. The *β* remains unaffected by either cycling damage or plasma power. In addition, to quantitatively assess the symmetry between LTP and LTD, we calculated an asymmetry factor defined as

10 $$\:A\:=\:\frac{\sum\:\varDelta\:{G}_{LTP}-\sum\:\left|\varDelta\:{G}_{LTD}\right|}{\sum\:\varDelta\:{G}_{LTP}+\sum\:\left|\varDelta\:{G}_{LTD}\right|}$$

where *A* = 0% indicates perfect symmetry. Using the measured data from Fig. 7(a) and 7(b), the calculated asymmetry factors were below 0.2% for all conditions, indicating excellent symmetry. However, the path-dependent asymmetry arising from differences in nonlinearity (β values, Fig. 7(e)) between LTP and LTD has a greater impact on neuromorphic system performance. This effect was explicitly considered in our simulations by incorporating separate *β* values for LTP and LTD [72].

To evaluate the impact of the measured device characteristics on hardware-based neuromorphic systems, convolutional neural network (CNN) simulations are conducted using the VGG11 architecture, as illustrated in the schematic diagram in Fig. 7(f) [73]. The network consists of multiple convolutional layers interleaved with MaxPooling layers to reduce spatial dimensions. It begins with a 3 × 3 convolutional layer with 64 channels, followed by MaxPooling. This is succeeded by two 3 × 3 convolutional layers with 128 channels, another MaxPooling layer, and two additional 3 × 3 convolutional layers with 256 channels, followed by MaxPooling. The network further includes two 3 × 3 convolutional layers with 512 channels, followed by another MaxPooling layer. Fully connected layers with 1024, 512, and 10 neurons are employed, and the output is classified using the Softmax function. ReLU activation and dropout are used to prevent overfitting and enhance generalization, enabling the classification of input images into 10 distinct classes [74]. By incorporating the device characteristics measured in Figs. 7(c–e), the effect of FTJs fabricated at different sputtering power levels is examined when used as synapses in the hardware neuromorphic systems. During forward propagation, read noise measured from the FTJs is reflected by adjusting the output current values as *I*_output_ = *I* · *N*(1, σ²), where σ represents the measured standard deviation of the read current. The synaptic weight ranges in the network were constrained by measured *G*_max_/*G*_min_ values, thus accurately incorporating device non-idealities into the simulation. Furthermore, the measured nonlinear potentiation and depression characteristics (*β* values) were explicitly incorporated into the synaptic-weight updates during backpropagation, as shown in Eqs. (7), (8) and (9). This approach enables an accurate evaluation of how sputtering-induced variations affect the performance of hardware-based neuromorphic systems [61, 75].

Figures 7(g) show the inference accuracy versus epoch graphs for FTJs in the pristine state (g-1) and damaged state (g-2). Each simulation is conducted 20 times, with the min-max accuracy shaded in light colors and the average accuracy represented by a solid line. Note that FTJ area of 3^2^ μm² is assumed to calculate the read noise sigma (Eq. 6). In Fig. 7(g-1), the pristine FTJs show that devices fabricated with higher plasma power achieve faster learning and higher final accuracy due to the larger *G*_max_/*G*_min_ values. Figure 7(g-2) shows the inference accuracy versus epoch for FTJs in the damaged state after cycling degradation. In this state, *G*_max_/*G*_min_ becomes similar for all plasma power levels (Figs. 7(b)), making *S*_*I*_/*I*² the dominant factor affecting inference accuracy. FTJs with lower plasma damage exhibit smaller *S*_*I*_/*I*², enabling better accuracy. Therefore, in the pristine state, *G*_max_/*G*_min_ is critical, favoring FTJs fabricated with higher plasma damage due to their higher TER ratios. However, in the damaged state, *S*_*I*_/*I*² becomes more important, making FTJs with lower plasma damage more effective as synaptic devices.

The relationship between FTJ area and inference accuracy is also examined. Area downscaling is essential for achieving high integration density in synaptic devices. However, FTJ read noise increases inversely with area (Eq. (6)), as experimentally demonstrated in previous studies [17, 18]. To verify the effect of increasing *S*_*I*_/*I*² with decreasing area and its impact on inference accuracy, simulations are conducted with varying FTJ areas and corresponding *S*_*I*_/*I*² values. In these simulations, nonlinearity and *G*_max_/*G*_min_ are assumed to remain constant, while *S*_*I*_/*I*² is scaled based on measured values for 100 μm² FTJs, proportional to the inverse of the area. This assumption is theoretically validated by Eq. (6) and supported by experimental data from prior studies [17, 18].

For sufficiently large FTJ dimensions (> 10^2^ µm^2^), the accuracy exceeds 90%, regardless of plasma power, since the small *S*_*I*_/*I*² has minimal impact. However, for smaller dimensions, increasing *S*_*I*_/*I*² reduces accuracy in the pristine state. Figure 7(h-1) shows that in the pristine state, higher plasma power results in less accuracy degradation during scaling. This is because FTJs fabricated with higher plasma power have larger *G*_max_/*G*_min_, making them less sensitive to the effects of increasing *S*_*I*_/*I*² as the area decreases. Therefore, FTJs fabricated with higher plasma power support lower area scaling limits for a given target accuracy. Conversely, in the damaged state (Fig. 7(h-2)), lower plasma power leads to less accuracy degradation due to scaling. After cycling damage, *G*_max_/*G*_min_ becomes similar across all plasma power levels, making *S*_*I*_/*I*² the dominant factor. FTJs with lower plasma damage exhibit smaller *S*_*I*_/*I*², making them less sensitive to area-induced noise effects. As a result, FTJs with lower plasma power achieve lower area scaling limits in the damaged state.

These simulation results clarify the relationship between plasma power and inference accuracy in neuromorphic systems, underscoring the importance of engineering fabrication parameters when designing FTJ-based neuromorphic systems. Specifically, the required number of synaptic conductance updates and network size should be considered to optimize the FTJ array fabrication process and determine appropriate sputtering power. In systems with a limited number of conductance updates, where the impact of read noise induced by plasma damage remains minimal, employing higher sputtering power is advantageous. This approach enhances the TER ratio and increases the on-current density, expanding the range of representable conductance levels and improving system performance. Conversely, in systems requiring frequent conductance updates, read noise caused by cycling-induced degradation becomes a critical limiting factor affecting overall system accuracy. In such cases, lower sputtering power should be used to minimize the formation of plasma-induced traps and suppress noise generation, even after extensive cycling operations.

By clarifying the relationship between plasma power, read noise, TER ratio, and current density while identifying the underlying physical mechanisms responsible for these behaviors, this study advances the understanding of FTJ device physics. Additionally, it offers valuable design guidelines for hardware-based neuromorphic systems, supporting more informed trade-off decisions between scalability, accuracy, and reliability for next-generation memory and computing applications.

## Conclusion

In this study, we systematically investigated the correlation between stochasticity and plasma-induced damage in FTJs, focusing on its impact on neuromorphic systems. We demonstrated that increasing plasma power during top electrode sputtering induces additional *V*_o_ in the HZO layer, forming shallow-level traps. These traps enhance the current magnitude through increased Poole-Frenkel emission while simultaneously amplifying read noise due to more frequent trapping-detrapping events. This dual effect highlights the complex interplay between increased current and noise caused by plasma damage. LFN spectroscopy, depth XPS analysis, and degradation energy characterization revealed that plasma-induced *V*_o_ traps are the primary contributors to stochasticity in FTJs. We found that while the fundamental degradation mechanism represented by the activation energy (*E*_a_) remains unaffected by plasma power, the initial state becomes increasingly prone to degradation as reflected by the degradation coefficient (C^*^).

Furthermore, neuromorphic system simulations using experimentally derived FTJ properties clarified that pristine FTJs fabricated with higher plasma power exhibit improved *G*_max_/*G*_min_ ratios and higher inference accuracy due to enhanced conductance levels. However, increased *S*_I_/*I*² after cycling-induced degradation reduces inference accuracy, indicating a critical trade-off between initial performance gains and long-term reliability.

This study clarifies the relationship between stochasticity and plasma-induced damage in FTJs, highlighting the underlying mechanisms governing their electrical performance and its implications on neuromorphic computing. We provided design guidelines for FTJ-based neuromorphic systems by emphasizing the trade-offs between conductance ratio, read noise, and device scalability. This understanding contributes to the development of more reliable and scalable ferroelectric-based neuromorphic hardware.

## Data Availability

Data will be made available on request.
